# Transjugular intrahepatic portosystemic shunt creation via isolated persistent left superior vena cava: a case series

**DOI:** 10.1186/s42155-020-00169-4

**Published:** 2020-10-06

**Authors:** Spencer B. Lewis, Guy E. Johnson, Karim Valji, Eric J. Monroe, Christopher R. Ingraham, Jeffrey Forris Beecham Chick, David S. Shin

**Affiliations:** 1 Division of Interventional Radiology, Department of Radiology, University of Washington, 1959 Northeast Pacific Street, Seattle, WA 98195 USA; 2 Division of Interventional Radiology, Department of Radiology, Seattle Children’s Hospital, 4800 Sand Point Way Northeast, Seattle, WA 9810 USA

**Keywords:** Left-sided superior vena cava, Persistent left superior vena cava, Isolated persistent left superior vena cava, PLSVC, Intrathoracic venous anomaly, Coronary sinus, Transjugular intrahepatic portosystemic shunt, TIPS

## Abstract

**Background:**

Isolated persistent left superior vena cava (PLSVC) is a rare vascular anatomic variant, which can be an incidental finding at the time of an endovascular procedure.

**Case presentation:**

This report describes the technical success, adverse events, and clinical outcomes of transjugular intrahepatic portosystemic shunt (TIPS) creation via isolated PLSVC. Three adult patients with cirrhosis and isolated PLSVC underwent TIPS placement successfully with one major adverse event. Two patients required TIPS revision within 90 days. There were no deaths within 90 days.

**Conclusions:**

TIPS creation via isolated PLSVC is feasible using standard techniques with a left jugular vein approach. Caution is warranted during the procedure to assess for any aberrant drainage pattern to the left atrium and to prepare for potentially challenging instrument navigation through the coronary sinus.

## Introduction

Persistent left superior vena cava (PLSVC) is the most common congenital intrathoracic venous anomaly, present in 0.1–2% of the general population (Sheikh and Mazhar 2014; Nagasawa et al. 2018). PLSVC typically occurs in conjunction with a normal or hypoplastic right SVC (Petridis et al. 2010a). Concomitant absence of the right SVC (i.e. isolated PLSVC) is a less common entity occurring in 0.01% of the general population (Sheikh and Mazhar 2014). In the absence of additional cardiac abnormalities, patients with isolated PLSVC are often asymptomatic, and the abnormality remains occult without thoracic imaging (Nagasawa et al. 2018).

The most common approach for creation of a transjugular intrahepatic portosystemic shunt (TIPS) is via the right internal jugular (IJ) vein, which allows access to the hepatic veins through the superior vena cava (Ratliff et al. 2006). Left IJ access has also been described, and potentially allows for greater engagement of the right hepatic vein, increasing the amount of torque that may be transmitted to the curved cannula for portal vein puncture (Ratliff et al. 2006). TIPS placement via an isolated PLSVC has been described in single case reports (Petersen and Clark 2008; Petridis et al. 2010b; Chovanec et al. 2010) and is associated with challenges, including an increased risk of arrhythmia, venous injury, and limited device maneuverability (Ratliff et al. 2006).

This report describes the technical success, adverse events, and clinical outcomes of TIPS creation via isolated PLSVC in three patients at a single academic institution.

## Case reports

This single center retrospective cohort study was conducted with *Institutional Review Board* approval and complied with the *Health Insurance Portability and Accountability Ac*t. Informed consent was not required for this retrospective study. All patients with an isolated PLSVC who underwent TIPS creation between January 1, 1995 to May 1, 2020 (304 months) were included. Patient demographics, indications, technical details, adverse events, and clinical outcomes are shown in Table 1. Technical success, defined as TIPS creation with post-intervention portosystemic gradient < 12 mmHg, was achieved in all cases. Adverse events were categorized according to the *Society of Interventional Radiology* (SIR) guidelines. Clinical success was defined as improvement or resolution of the original procedure indications. Death within 90 days was recorded for mortality.

**Table 1:** Patient demographics, indications, technical details, adverse events, and clinical outcomes

Case #	1	2	3
**Etiology of Cirrhosis**	Alcohol	HCV	HCV
**Indication**	Portal Hypertension Treatment Prior to Major Abdominal Surgery	Refractory Ascites	Recurrent Hydrothorax
**Child-Pugh Class**	B	B	C
**Known PLSVC**	No	No	Yes
**Intraoperative Guidance for Portal Vein Access**	CO2 Portography	IVUS	IVUS
**Stents Used**	Viatorr 12-mm × 6-cm / 2-cm, Wallstent 12-mm × 6-cm	Viatorr 10-mm × 6-cm / 2-cm	Viatorr 8–10-mm × 8-cm / 2-cm
**Pre-TIPS Portosystemic Gradient (mmHg)**	18	20	12
**Post-TIPS Portosystemic Gradient (mmHg)**	7	4	4
**Fluoroscopy Time (min)**	81.2	20.8	29.1
**Radiation Dose (mGy)**	1978	506	708
**Technical Success**	Yes	Yes	Yes
**Complications**	Intraperitoneal Hemorrhage Requiring Transfusion and Angiography (Class D)	None	None
**Revisions**	POD 1 for stenosis on angiography	None	POD 31 for elevated velocities on Duplex
**Follow-up Duration (outcome)**	924 days (transplant, lost to follow-up)	242 days (death)	363 days (alive with persistent hydrothorax)

*POD* Post-operative day

### Case 1

**Fig. 1:**
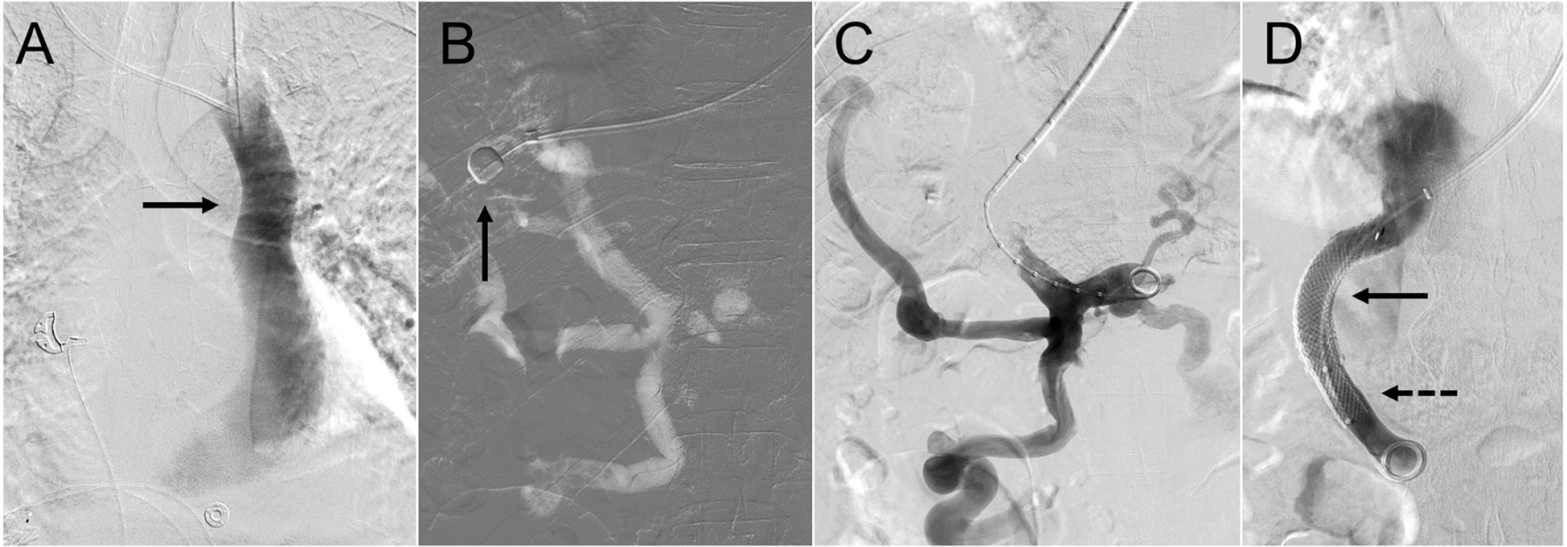
A middle-aged man with end-stage liver disease, portal hypertension, colovesical fistula, and isolated persistent left superior vena cava. **a** Left internal jugular venography demonstrating the isolated persistent left superior vena cava (solid arrow). **b** Wedged portal venography using an occlusion balloon (solid arrow). **c** The right portal vein was accessed using a Rösch-Uchida needle, and portal venography was performed. A 12-mm × 6-cm (covered) / 2-cm (uncovered) Viatorr stent-graft was deployed. Post-deployment portal venography; however, showed limited flow. **d** After Viatorr stent-graft (solid arrow) deployment, an additional 12-mm × 6-cm Wallstent (dashed arrow) was placed to extend the portal aspect of the construct, resulting in brisk in-line flow

Patient 1 is shown in Fig. 1. A middle-aged man with end-stage liver disease (ESLD) secondary to alcohol use, who was undergoing liver transplantation (LT) evaluation, presented with a colovesical fistula secondary to diverticulitis. A decision was made to repair the fistula before LT. TIPS placement was performed for treatment of portal hypertension prior to the planned fistula repair to reduce associated perioperative risk.

After failure to access the SVC through the right IJ vein, the left IJ vein was accessed. Left IJ venography demonstrated an isolated PLSVC (Fig. 1a). A standard 10-French × 40 cm sheath (Rösch-Uchida Transjugular Liver Access Set; Cook; Bloomington, IN) was advanced, through the coronary sinus, without arrhythmia, into the right hepatic vein. Balloon-occlusion carbon dioxide portal venography (Fig. 1b) was performed. The right portal vein was targeted using a 14-gauge Rösch-Uchida needle. Portal venography (Fig. 1c) was then performed. A 12-mm × 6-cm (covered) / 2-cm (uncovered) Viatorr stent-graft (W. L. Gore & Associate; Newark, DE) was deployed. The stent was dilated using a 12-mm high-pressure angioplasty balloon. Post-deployment portal venography, however, showed limited flow, likely due to inadequate extension of the stent into the portal system. Therefore, a 12-mm × 6-cm Wallstent endoprosthesis (Boston Scientific; Marlborough, MA) was deployed to extend the caudal end of the stent into the main portal vein. The entire stent construct was then dilated using a 12-mm high-pressure angioplasty balloon. Final portal venography showed brisk in-line flow (Fig. 1d) through the TIPS.

Four hours following TIPS placement, the patient became hypotensive. Computed tomography with contrast demonstrated intraperitoneal hemorrhage. Hepatic arteriography, however, showed no arterial extravasation, and the patient stabilized. TIPS venography, performed in the same setting, showed a > 50% stenosis. Angioplasty was performed using a 12-mm balloon.

The patient subsequently developed hepatorenal syndrome and was prioritized to undergo LT. Successful LT with diverting colostomy creation occurred 38 days later.

### Case 2

A middle-aged man with ESLD secondary to hepatitis C virus (HCV), being evaluated for LT, presented with refractory ascites.

**Fig. 2:**
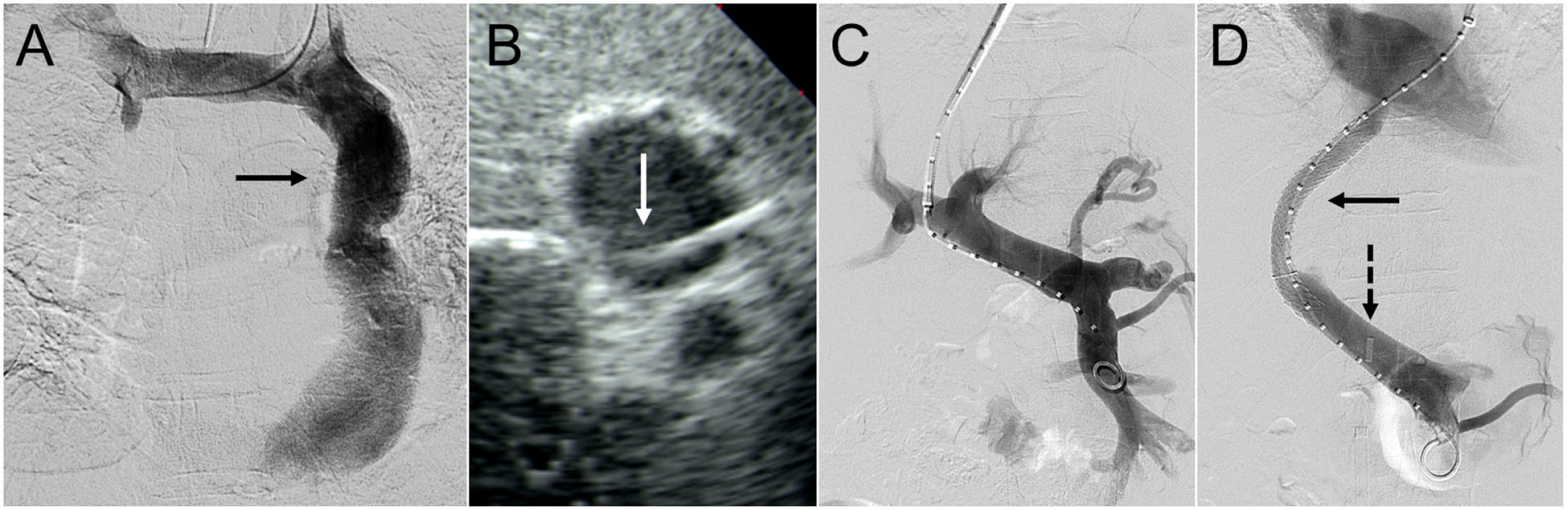
A middle-aged man with end-stage liver disease, hepatitis C, recurrent ascites, and isolated persistent left superior vena cava. **a** Left internal jugular venography demonstrating the isolated persistent left superior vena cava (solid arrow). **b** Perpendicular-projecting intravascular ultrasound-guided access to the right portal vein (solid arrow). **c** Portal venography was performed. **d** A 10-mm × 6-cm (covered) / 2-cm (uncovered) Viatorr stent-graft was deployed (solid arrow) resulting in widely patent TIPS. A perpendicular-projecting intravascular ultrasound probe (dashed arrow) is shown

The left common femoral vein was accessed and a perpendicular-projecting intravascular ultrasound (ACUSON AcuNav, Johnson & Johnson Medical; New Brunswick, NJ) was introduced. After failure to gain access to the right atrium through the right IJ vein, the left IJ was accessed. Left IJ venography demonstrated an isolated PLSVC (Fig. 2a). A standard 10-French × 40 cm sheath was advanced through the coronary sinus and right atrium without arrhythmia, and then into the middle hepatic vein. A 14-gauge Rösch-Uchida needle was advanced into the right portal vein under real-time intravascular ultrasound guidance (Fig. 2b). Portal venography (Fig. 2c) was then performed. A single 10-mm × 8-cm (covered) / 2-cm (uncovered) Viatorr stent-graft was deployed and dilated using a 10-mm high-pressure angioplasty balloon. Post-deployment portal venography showed brisk in-line flow through the TIPS (Fig. 2d).

Follow-up computed tomography with contrast showed a patent TIPS with reduction in ascites. However, the patient died from liver disease prior to LT on post-operative day 242.

### Case 3

**Fig. 3:**
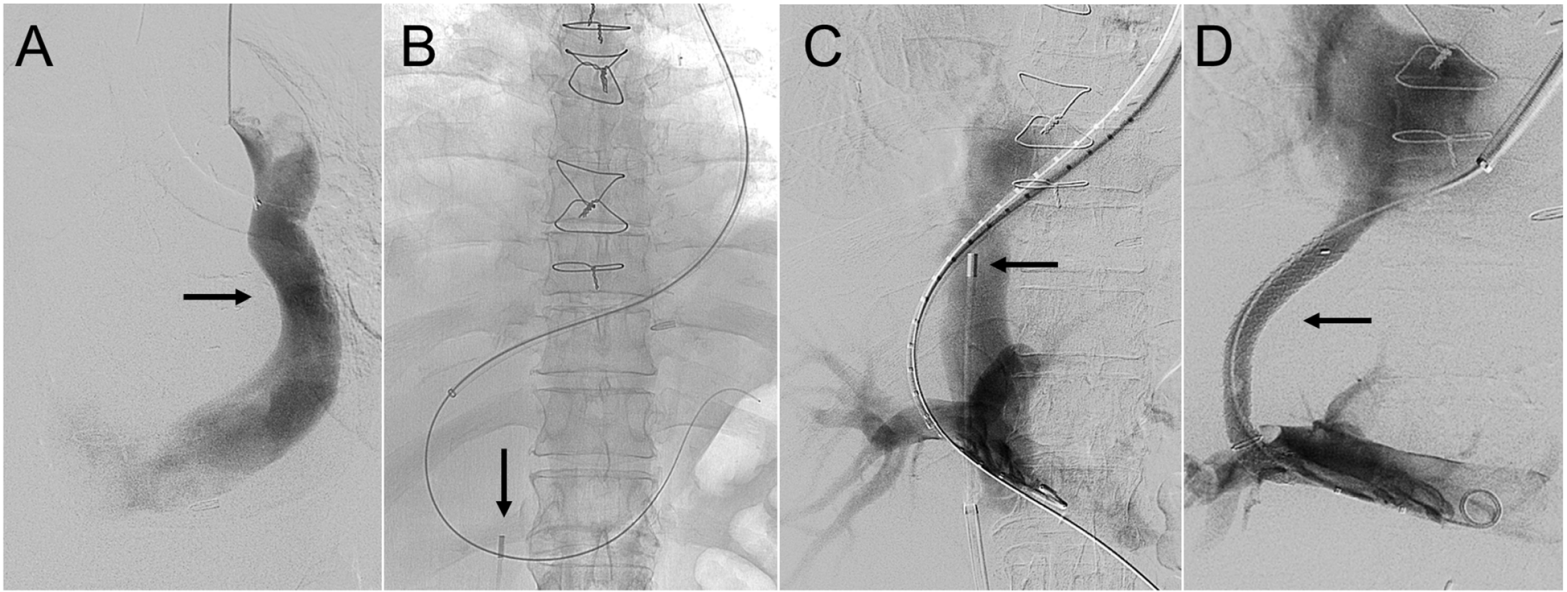
A middle-aged man with hepatic hydrothorax and isolated persistent left superior vena cava. **a** Left internal jugular venography demonstrating the isolated persistent left superior vena cava (solid arrow). **b** Access from the left internal jugular vein into the portal system via the left superior vena cava and coronary sinus using intravascular ultrasound-guidance (solid arrow). **c** The right portal vein was accessed with a Rösch-Uchida needle, and simultaneous portal and caval venography was performed. A perpendicular-projecting intravascular ultrasound probe (solid arrow) is shown. **d** An 8–10-mm × 8-cm (covered) / 2-cm (uncovered) Viatorr controlled expansion stent-graft (solid arrow) was deployed with resultant brisk in-line flow

A middle-aged man with hepatic hydrothorax presented for TIPS creation. The left common femoral vein was accessed, and a perpendicular-projecting intravascular ultrasound was introduced. Left IJ venography confirmed a known isolated PLSVC (Fig. 3a). A 10-French sheath was advanced through the coronary sinus and right atrium, with transient tachyarrhythmia, into the right hepatic vein (Fig. 3b). A standard 14-gauge Rösch-Uchida needle was advanced into the right portal vein using intravascular ultrasound guidance. Portal venography (Fig. 3c) was then performed. A single 8–10-mm × 8-cm (covered) / 2-cm (uncovered) Viatorr endoprosthesis with controlled expansion was deployed. The stent was dilated to 8-mm. Post-deployment portal venography (Fig. 3d) showed brisk flow through the TIPS.

The patient continued to have hepatic hydrothorax, and TIPS revision was performed using 10-mm angioplasty. Revision was performed at an outside institution and procedural details were not available. Subsequent TIPS ultrasound revealed a patent TIPS with normal velocities (150–190 cm/s) (Fig. 3f).

## Discussion

A TIPS may be placed in the setting of congenital or acquired anatomic challenges using various methods, including direct transcaval portal access (i.e. direct intrahepatic portocaval shunt) (Petersen and Clark 2008), transfemoral intrahepatic routes for challenging transjugular approaches, and left-sided access in patients with situs inversus (Chovanec et al. 2010; Postoak et al. 2002; Kundaragi and Ganjoo 2018). Left IJ access, while performed less commonly during TIPS in patients with standard anatomy, may provide trajectory benefits in patients with cirrhosis and cranially situated livers with failed previous right IJ access attempts (Hausegger et al. 1998).

Echocardiography is often performed prior to TIPS placement, particularly in patients with known diastolic dysfunction or pulmonary arterial hypertension, as these are predictors of increased post-procedural mortality (Cazzaniga et al. 2007; Copelan et al. 2014). While all patients in this series underwent echocardiography prior to TIPS placement, an isolated PLSVC was only identified in one patient. Follow-up echocardiography was performed in two patients after TIPS placement (and angiographic diagnosis of isolated PLSVC) for the pre-transplantation evaluation. While one of these studies demonstrated a dilated coronary sinus typical of PLSVC, the diagnosis was not suggested in either echocardiography evaluation, suggesting that this anatomic variant is not reliably diagnosed on conventional echocardiography.

As was the case in two of the patients in this study, the diagnosis of isolated PLSVC may be new and incidental at the time of TIPS procedure. In these cases, cavography must be performed to carefully assess the anatomy. If the PLSVC drains into the left atrium, TIPS would not be feasible from the jugular vein access, and femoral access may be considered. If the PLSVC is associated with an unroofed coronary sinus or atrial septal defect, as occurs in 50–70% of cases (Tak et al. 2002), the procedure should be aborted as passage of the sheath may worsen the existing defect and TIPS creation may lead to right to left shunting and embolism. In cases of equivocal venography, TIPS placement may be deferred until dedicated cardiac imaging is performed to delineate the anatomy. If no aberrant drainage to the left atrium is noted, TIPS placement may proceed, as in these three cases, with the caveat that the procedure may be more technically challenging than a standard TIPS placement.

TIPS creation via isolated PLSVC poses potential technical challenges related to the broad angulation of the access sheath across the heart. This sheath trajectory may lead to difficulty in obtaining a favorable torque angle for portal vein access and also may limit the “pushability” of the sheath across the hepatic parenchymal tract. In order to mitigate these challenges, the authors suggest using intravascular ultrasound guidance to visualize the portal vein target and needle trajectory in real time, accentuating the curve of the Rösch-Uchida needle metal cannula to optimize “torquability” and aim, and utilizing an extra stiff guide wire to aid in tract dilation and sheath advancement.

A perpendicular-projecting intravascular ultrasound has become widely adopted for adjunct intraprocedural imaging-guidance during TIPS and has been shown to reduce the number of needle passes, increase technical success, and decrease fluoroscopy time (Farsad et al. 2012; Pillai et al. 2016; Kao et al. 2016). The first case in this series, which was performed without intravascular ultrasound, was prolonged, required multiple needle passes, and resulted in intra-abdominal hemorrhage. It is possible that the lack of complementary ultrasound guidance contributed to the technical challenges encountered, especially in the setting of PLSVC. Transabdominal ultrasound is another option for portal venous access guidance and also has been shown to reduce procedure times and radiation doses (Cam et al. 2020; Tavare et al. 2017), and likely more readily available and cost effective than intravascular ultrasound.

Transient and asymptomatic tachyarrhythmias are not uncommon during wire and catheter manipulation through the lower PLSVC and across the coronary sinus (Postoak et al. 2002). Close communication with the anesthesiologist is paramount. The trajectory across the heart must be visualized under real-time fluoroscopy when the sheath is being advanced through the hepatic parenchymal tract as bowing and buckling of the sheath may occur within the enlarged coronary sinus.

Of note, two of the three patients in this series required TIPS revision within the first 3 months of creation. While this is a small sample size, the revision rate is higher than the 29% suggested by Perello et al. (2019), possibly because the technical challenges associated with this aberrant anatomy predispose patients to early shunt malfunction. Further study is necessary to determine whether this finding holds in a larger patient series.

This study is limited by its retrospective design and small sample size at a single tertiary care institution. As a result, the positive outcomes seen in this study may not be reflective of the safety profile of TIPS placement in this patient population. Significant arrhythmias during instrumentation through the coronary sinus and right atrium are possible. Experienced operators familiar with this anatomic variant and its drainage patterns may proceed. Alternatively, direct intrahepatic portocaval shunt creation (DIPS) may be considered via a transfemoral approach to avoid working within the aberrant anatomy.

## Conclusion

TIPS creation via isolated PLSVC is feasible using standard techniques with a left jugular vein approach. The presence of isolated PLSVC may be previously unknown. Caution is warranted during the procedure to assess for any aberrant drainage pattern to the left atrium and to prepare for potentially challenging instrument navigation through the coronary sinus.

## Data Availability

Not applicable.

## Abbreviations

PLSVC: Persistent left superior vena cava
SVC: Superior vena cava
TIPS: Transjugular intrahepatic portosystemic shunt
IJ: Internal jugular
SIR: *Society of Interventional Radiology*
ESLD: End-stage liver disease
OLT: Orthotopic liver transplantation
HCV: Hepatitis C virus
